# Interactive Informed Consent: Randomized Comparison with Paper Consents

**DOI:** 10.1371/journal.pone.0058603

**Published:** 2013-03-06

**Authors:** Michael C. Rowbotham, John Astin, Kaitlin Greene, Steven R. Cummings

**Affiliations:** 1 California Pacific Medical Center Research Institute, San Francisco, California, United States of America; 2 San Francisco Coordinating Center, San Francisco, California, United States of America; University of Warwick, United Kingdom

## Abstract

Informed consent is the cornerstone of human research subject protection. Many subjects sign consent documents without understanding the study purpose, procedures, risks, benefits, and their rights. Proof of comprehension is not required and rarely obtained. Understanding might improve by using an interactive system with multiple options for hearing, viewing and reading about the study and the consent form at the subject’s own pace with testing and immediate feedback. This prospective randomized study compared the IRB-approved paper ICF for an actual clinical research study with an interactive presentation of the same study and its associated consent form using an iPad device in two populations: clinical research professionals, and patients drawn from a variety of outpatient practice settings. Of the 90 participants, 69 completed the online test and survey questions the day after the session (maximum 36 hours post-session). Among research professionals (n = 14), there was a trend (p  = .07) in the direction of iPad subjects testing better on the online test (mean correct  =  77%) compared with paper subjects (mean correct  =  57%). Among patients (n = 55), iPad subjects had significantly higher test scores than standard paper consent subjects (mean correct  =  75% vs 58%, p < .001). For all subjects, the total time spent reviewing the paper consent was 13.2 minutes, significantly less than the average of 22.7 minutes total on the three components to be reviewed using the iPad (introductory video, consent form, interactive quiz). Overall satisfaction and overall enjoyment slightly favored the interactive iPad presentation. This study demonstrates that combining an introductory video, standard consent language, and an interactive quiz on a tablet-based system improves comprehension of research study procedures and risks.

## Introduction

### Informed consent is the cornerstone of human research subject protection

Research studies are growing in complexity and duration. Concerns persist about the adequacy of the consent process, in particular whether subjects are fully informed and actually comprehend the study. The consent form is a detailed – and generally long – description of the protocol, possible benefits and harms, study procedures, and patient rights. Most often, the first step is giving a paper consent form to the potential subject to review. Staff and the study investigator may assist the potential subject in reviewing the consent form and answering questions, but this may be insufficient and is generally not standardized. Many subjects then sign – and thereby provide consent – without understanding the study purpose, procedures, risks, benefits, and their rights. Documentation of comprehension is not required and rarely obtained before a subject is allowed to sign the form. The standard process of informed consent is risky because the subject may have complications that he or she did not realize were inherent in the research or procedure. Misunderstandings can become contentious.

### Features of an ideal informed consent process

Understanding may be improved by providing potential subjects with several options to hear, view or read the consent form at their own pace. If done interactively, subjects can be given immediate feedback about their level of understanding of study procedures, risks, and so on, thereby theoretically increasing their overall comprehension. A test can then verify comprehension of key study elements before the subject is allowed to sign the ICF and enter the study.

### The need for a comparative study

There has been little study of interactive methods to improve consent. A few studies have tested informed consent on computers – with colorful graphics and multimedia – and compared them with written documents before elective surgeries with mixed results about whether understanding and satisfaction were improved [1]. Furthermore, the consent forms evaluated were for surgery or single procedures, not complex multi-visit clinical research studies that present the greatest risk of an inadequate consent process. There have been no studies of whether an interactive process, including home-based learning, audiovisual options, and the requirement to complete an interactive quiz improves understanding of the study or procedure.

### Interactive Consent System

An online interactive consent system (Mytrus, San Francisco) has been developed with several features that might improve potential subjects’ understanding of the study, including video and audio summaries of the consent form with options to read the document online or in printed form. The consent process in this system includes an interactive quiz.

This prospective randomized study compared the IRB-approved paper ICF for an actual clinical research study with an interactive presentation of the same study and its associated ICF using an iPad device in two populations: clinical research professionals, and patients drawn from a variety of outpatient practice settings. Outcome measures included comprehension, delayed recall, and ratings of user acceptability of the standard paper ICF or the interactive consent process (as delivered via an iPad and including a comprehension quiz).

## Materials and Methods

### Overview

The prospective randomized study, approved by the Institutional Review Board (IRB) of California Pacific Medical Center, was conducted in two parts:

In Part 1, clinical research professionals (primarily IRB panel members) at California Pacific Medical Center Research Institute who agreed to participate were randomized by computer to learn about a chemotherapy neuropathy clinical research study by reviewing either the IRB-approved standard paper consent form or the same consent form via an iPad based interactive system. After orientation to the study by a research coordinator, the session took place in the subject’s office and could last up to one-hour. Each participant completed an online survey the next day (minimum 18 hours post-session) that consisted of a test of delayed recall, included questions about the user experience, and free text space for comments. Participants had to complete the online survey within 36 hours of the study session.

In Part 2, patients drawn from a variety of clinical practices at CPMC who agreed to participate were randomized by computer to learn about the same clinical research study by reviewing either the IRB-approved paper consent form or via the same iPad based interactive system used for Part 1. The session took place after they finished their medical appointment in either a separate room or a quiet area of the waiting room. To be eligible, participants had to be between age 18–80, literate in written and spoken English, have internet access available to complete the online survey the next day, be able to stay after their medical visit is completed for the one-hour study session. Participants could not have cancer or have undergone cancer chemotherapy. As in Part 1, each participant completed an online survey the next day that tested delayed recall and includes questions about the user experience. Participants had to complete the online survey within 36 hours of the study session.

### Consent form used to assess the interactive system

The complete consent form from a previously approved longitudinal study in patients with cancer undergoing chemotherapy of the possible nerve damaging effects of chemotherapy (‘chemotherapy neuropathy study’) was used because it provided the needed level of complexity required for assessing how an interactive consent would perform when used for subjects enrolling in an actual trial. The purpose of the chemotherapy neuropathy study was to evaluate the development of peripheral neuropathy (a problem with the nerves that carry information between the brain and spinal cord and the rest of the body that can produce pain, loss of sensation, and an inability to control muscles) and neuropathic pain (chronic pain caused by nerve damage) in patients undergoing chemotherapy. The chemotherapy neuropathy study did not involve an intervention or invasive procedures. The three study visits over 6 months relied on questionnaires, simple bedside sensory testing, and quantitative sensory testing (QST) to look at changes in peripheral nerve function over time in patients receiving chemotherapy. The consent form included relatively standard information about compensation and outcomes of study-related injury or harm to subjects was included in the consent form as follows: “Investigators have no special program to provide compensation if injury occurs during biomedical research. If you are injured or made ill as a result of participation in this study, treatment will be made available. Because insurance companies may not pay for research-related costs, they may not pay for an injury resulting from your participation in this study. Any costs not paid by your insurance company will be your responsibility.”

### Session procedures

With IRB approval, an informational letter in lieu of signed informed consent was used in order to maintain participant anonymity. The informational letter contained all required consent elements including the study purpose (that the study is designed to test their comprehension and recall of a consent form that could be used in a clinical research study), the study design, what will be asked of the participant if he/she agrees to participate, risks of participation, the voluntary nature of participation, and compensation ($27 gift card). The study was completely anonymous; no personally identifying information was recorded on any study documents.

Interested subjects were then given the iPad and asked to enter the following information: their assigned subject username, year of birth, gender, and the registration number on their gift card for reimbursement purposes. The iPad then randomly assigned the subject to either the paper or interactive iPad group in a ratio of 1:1. The paper group returned the iPad and received a paper consent form for review; the iPad group proceeded with the electronic interactive version of the consent. The interactive version began with an informational video outlining the main features of the study, then presented a series of screens of the same consent form as in the paper participants (in the same font), followed by quiz questions at the end of the chemotherapy neuropathy consent form.

Participants were encouraged to fully review the consent form before signaling to the research coordinator when they felt they had completed their review to a sufficient degree that they would actually sign (or are sure they would refuse to sign) the consent form to participate in the cancer chemotherapy study. The iPad recorded the amount of time spent on the device and the number of correct/incorrect questions. The study coordinator recorded the number of minutes spent reviewing the consent for the paper group.

Participants randomized to the interactive iPad consent were encouraged to complete the quiz that followed the consent form. The quiz consisted of 7 multiple-choice questions covering the following areas: (1) the main reason for the study; (2) the role of the study doctor; (3) what happens ‘if you agree to participate’; (4) risks of quantitative thermal sensory testing; (5) reimbursement for participation; (6) what happens ‘if you were accidentally injured’; (7) total duration of the study. Each multiple-choice question had 4 possible answers. If the participant marked the wrong answer on the iPad, the device would signal the answer was incorrect and return to the screen on the consent relevant to that particular question before allowing the subject to answer again. Participants had three chances to answer correctly before the device moved to the next question.

At the end of the session, the study coordinator provided an information sheet with the participant’s subject ID, the website URL for accessing the followup online questionnaire, time window for online questionnaire completion, and contact information for the study coordinator in case the participant has questions or problems accessing the online survey. Participants were also given their gift card and notified that the gift card had no value until the online questionnaire was completed and that lost or stolen gift cards could not be replaced due to the anonymous nature of the study. The exact time window for completion of the online questionnaire was reviewed. Participants were told that they could not complete the online survey until the next day and if they did not complete the survey within a 36 hour window they would no longer be able to access the website and would not be compensated. In actuality, the gift cards were already activated to ensure that all participants would be able to use them as soon as they completed the online survey. Once the subject has completed the online questionnaire, their participation in the study was complete.

The web survey began with questions about age, gender, years of education, and race/ethnicity. Twelve multiple-choice comprehension and recall questions were then asked, summarized as follows:

1. main reason for the study
2. if you think you have had an injury as a result of the study, whom should you call?
3. if you have questions about your rights as a participant in this study, who should you call?
4. if you decide to participate, then which of the following will happen?
5. which statement about quantitative thermal sensory testing is true?
6. risks of quantitative thermal sensory testing
7. if you were accidentally burned during the study and needed treatment, which is true?
8. if you agree to participate, which one of the following will you receive?
9. total duration of study
10. which one of these statements is true? (you will have QST; study doctor may prescribe treatments for painful neuropathy that develops during the study; if you stop chemotherapy, you will not need to have further visits for this study; results of your QST will be sent to your doctor)
11. during the quantitative thermal sensory testing procedure, you will be asked to: (continue until probe feels painfully cold; push button whenever you want probe to return to neutral temp; rate the heat sensation 0–100; all of above)
12. which of the following statements is true? (if you agree to participate you must complete all 3 exams; you may stop for any reason at all; if you stop before final you will not receive payment for participating; none of the above)

Five of the questions (1,4,6,8,9) were duplicates of the questions appearing in the iPad quiz. Participants were then asked about their willingness to participate in the cancer chemotherapy study if they actually had cancer and were an eligible subject; user experience questions; and a free text box for likes/dislikes/problems they perceived with the system they were assigned to (paper or interactive).

### Methods of Data Analysis

The target sample size was set at a low number (N  =  15) for Part 1 because the aim of Part 1 was to pilot-test the two presentations of the consent document (paper and electronic/tablet-based) for differences in comprehension and collect data on the functionality of and user experience with the interactive iPad consent. The sample size for Part 2 was determined based on an estimated 20% difference in comprehension scores between those randomized to the standard written consent and subjects receiving the interactive iPad consent. With 27 subjects per group (N  =  54), there would be 95% power to detect this level of difference at the p < .05 level. Participants who failed to complete the follow-up online survey provided no evaluable data and were not included in the statistical analysis. Estimating a 25–30% failure rate in completing the follow-up online survey, a target sample size of 75 participants for Part 2 was set. Depending on the outcome measure, chi-squared, unpaired t-test, and Wilcoxon signed ranks tests were used to determine if significance was present at the p < .05 level.

## Results

### Participation

In Part 1, 15 subjects agreed to participate and 14 completed the followup online survey. In Part 2, 75 subjects agreed to participate and 55 completed the online survey. Of Part 2 participants, 66% were female. The mean age was 50 (18–35: 18%; 36–55: 35%; 56–80: 57%). Overall, the Part 2 group was well educated: 43% had a college degree and another 30% had an advanced degree (only 4% had no education following high school diploma). Consistent with San Francisco’s population, the largest racial/ethnic groups were Caucasian (76%) and Asian (17%).

### Online survey results (Table 1)

Table 1 provides a breakdown of results by question for each group.

**Table 1:** Post-review online survey test question results.

	group1	group 1	group 2	group 2
Question	iPad	Paper	iPad	Paper
Q1 (Reason for study) *	88	67	69	72
Q2 (Who to call if injured)	100	83	89	72
Q3 (Who to call if questions)	38	33	15	14
Q4 (Continue with normal treatments) *	75	33	89	62
Q5 (What involved in QST)	50	67	77	55
Q6 (Risks of QST) *	100	50	77	48
Q7 (If you require treatment)	88	67	96	59
Q8 (Amount of compensation) *	100	83	96	76
Q9 (Duration of Study) *	88	33	96	59
Q10 (What involved in study)	75	83	69	72
Q11 (What involved in QST)	38	0	42	21
Q12 (Free to stop participating any time)	88	83	85	86
**OVERALL PERCENT CORRECT**	77	57	75	58

* denotes questions also appearing on the iPad quiz at the time of review. Group 1 (n = 14) overall % correct p<0.07 in favor of iPad. Group 2 (n = 55) overall p <0.001 in favor of iPad. Combined overall difference P<0.001 in favor of iPad.

Part 1: Among the 14 research professionals, comparing iPad vs. Paper groups, there was a definite trend (p  = .07, chi-squared test) in the direction of iPad subjects scoring better (mean correct  =  77%) compared with paper (mean correct  =  57%). For the questions that were duplicates of the quiz presented on the iPad, the differences were greater. Participants randomized to the iPad were correct 90.2% of the time on the five duplicated questions, and correct only 68.1% of the time for the seven non-duplicated questions. In contrast, participants randomized to review the paper consent scored similarly for duplicated and non-duplicated questions (53.2% and 59.4%). Interestingly, although the research professionals scored very well on questions related to compensation, they scored poorly on technical questions related to quantitative sensory testing (the main procedure being used to detect nerve damage) at 38% correct in the iPad group and zero% correct in the paper group.

Part 2: Among the 55 patients, comparing iPad vs. Paper groups, there was a significant difference (p < .001, chi-squared test) in total score on the online next day survey with iPad subjects scoring better (mean correct  =  75%) compared with standard paper consent (mean correct  =  58%). Participants randomized to the iPad did much better than paper on the five duplicated questions (85.4% vs 63.4%). However, the iPad group also outperformed the paper group to nearly the same extent on the seven non-duplicated questions (67.6% correct vs 54.1% correct).

### Time spent reviewing the consent form

Considering subjects in both parts of the study, the total time spent reviewing the paper consent was 13.2 minutes (range 4.0–21.5). Subjects randomized to the iPad spent an average of 22.7 minutes total (range 12.4–50.4) on the three components to be reviewed, significantly more than the paper subjects (p<.001, unpaired t-test). In the iPad subjects, a mean of 445 seconds (7.4 minutes) was spent viewing the video (three subjects watched it twice), 685 seconds (11.4 minutes) were spent reviewing the consent document, and 235 seconds (3.92 minutes) were spent on the quiz (range 2–6 minutes).

There was no significant correlation between time spent reviewing the consent form and comprehension as measured by % correct responses in the online survey (whether looking at groups together or separately).

### User experience and user feedback questions

The research professionals in Part 1 who were randomized to the iPad reported slightly greater overall satisfaction on the 10 point scale (7.5 vs. 7.0), and iPad users reported slightly more enjoyment/interest (2.3 vs 1.7 on the 4 pt. scale). Only two participants provided feedback related to the consent form, writing "The consent form is overloaded with information. The way definitions of medical terms are incorporated is very awkward." Another wrote “I was randomized to the iPad however in reality would always prefer a hard copy of any ICD so that I have it for reference and if I was a patient I could take it away with me and share it with family members etc.”

In Part 2, 62% of iPad subjects said they would participate in the study compared with 69% of paper-based subjects (p = NS). Stated reasons for non-participation in order of frequency were: “worry about time” (25%); “lack of interest” (21%); “privacy concerns” (17%); “concern about QST pain” (12%); and, “skin biopsy” (4%). In terms of user experience, the differences were also not statistically significant. There was a trend in the direction of greater overall satisfaction on the 10 pt. scale for iPad users (7.7 vs. 6.5). Differences between reported enjoyment and understandability of the consent were not significant.

User feedback for Part 2 was noteworthy for concerns about the amount and way information is presented in the consent form (“overloaded with information”, “babble of words”, “boring and made me not want to read the details”). A few subjects randomized to the paper version wished they had had the opportunity to see the iPad version (“Illustrations and interactive programs would be much smarter and a better way to explain what is really going to occur. A form is just a form and shouldn't be.”). Multiple users asked for a bullet point format with access to background information if more was desired. User feedback in Part 2 was also noteworthy for concerns about who would pay for treatment of any injury related to the study. Some subjects felt very strongly that there should not be any possibility of a research subject being financially responsible for care in the event of an injury. A few subjects were concerned about the possible response of their health insurer (“Would they have a negative response to my participating with the study?”).

When all participants were combined, the differences in overall satisfaction rating favoring the iPad approached significance (p  =  .09, Wilcoxon signed-ranks test). The iPad users reported slightly greater overall enjoyment (3.2 vs. 2.9; p<0.05, Wilcoxon signed-ranks test), but self-reported differences in comprehension of the consent were not significant.

## Discussion

A major issue in human subjects research is the quality of the consenting process. IRBs and external monitors are able to audit whether all the paper consent forms are signed, but unless additional steps are taken to ensure comprehension the quality of the interaction between research personnel and potential subjects is unknown. Riecken and Ravich’s 1982 study examined informed consent in a group of research patients in Veterans Administration hospitals [2]. Even though signed consent forms were on file, 28% did not know they were research subjects. Readability analysis of the consent forms indicated a college education was necessary for comprehension, and also found subjects were more likely unaware of their participation when someone other than the investigator took exclusive responsibility for explaining the study. Three studies have examined how long research subjects actually spend ‘reading’ consent documents before signing. Baren and colleagues conducted a study in the emergency department of the informed consent process for an intimate partner violence survey [3]. A research assistant first gave a scripted verbal description of study. Of those agreeing to participate (1,312 or 82%), only about half (53%) read the brief (2 pages, 11 paragraphs) consent form at all. Of those that did read the consent form, the majority spent less than one minute reviewing it before signing. The study by McNutt and colleagues provides a similar example [4]. After providing information verbally to two samples of women, research assistants observed that over half of the women read their consent forms in thirty seconds or less before signing. Lee and colleagues gave 73 subjects a comprehension quiz right after the consent form was signed [5]. Of six elements of the form, about half the subjects were not aware of two or more study-related risks. Actual time spent reading the consent form was a mean of 2 minutes, less than the estimated 4–5 minutes needed based on standard reading rates for high school students and a 1300 word consent.

In their systematic review, Flory and Emanuel raised the distinction between rote memorization of the content of the ICF and true understanding of the research study [6]. Of twelve trials of multimedia interventions (computer or video in place of or in addition to the written ICF), only three significantly increased understanding. They concluded that for most of the trials of enhanced consent forms that showed a significant effect, the simulation of the consent process was unrealistic in that there was no active discussion, only a reading of the form. In such a setting, the form becomes the participant’s only source of information and this exaggerates the impact of changes to the form. In research, the informed consent process is already formalized through federal regulations that require a written consent form, and video- based and computer-based interventions may not add much to this relatively thorough disclosure process. In contrast, the five trials with testing and/or feedback all improved apparent understanding. Flory and Emanuel also pointed out the strong links between higher educational level, higher reading level, and higher understanding scores. The 2009 study by Tait and colleagues showed that baseline knowledge and younger age, as well as the use of an interactive video in the setting of non-research cardiac procedures were associated with ‘complete’ understanding of the ICF [1].

The recent commentary by Schenkel and Meisel elaborated three practical issues to be considered in improving comprehension of ICFs [7]. First, more is not always better. More information in consent forms may produce the opposite effect of the subject spending even less time reviewing the form before signing. Second, timing matters – in both clinical practice and research the informed consent process may take place immediately before a procedure, after the patient or research subject is already psychologically committed to proceed and the optimum time for weighing risks and benefits has passed. Third, technology can help. Strategies that do not involve physicians (because they are often in a rush), such as an interactive consent aided by a nurse or other educated health care professional, are needed. However, the physician-investigator must play an important role as the study by Riecken and Ravich showed the subjects were “more likely unaware” when someone other than investigator took exclusive responsibility for explaining the study.

Creative, non-technological interventions continue to advance. Antoniou and co-workers attempted to ensure subjects would stay engaged by constructing a three level patient information sheet for an online survey study [8]. The first level was brief but provided enough information for most to make a decision; the second level corresponded to a ‘standard’ patient information sheet. Of the 552 participants, 77% went to first level (23% never accessed the information sheet); only 18% went to the second or third level. Fink and colleagues asked patients to recount what they had been told in the informed consent to confirm understanding the key risks of the (non-research) surgical procedure they were to undergo [9]. In this large study of 575 patients, there was a small but significant effect on comprehension compared to subjects who were consented without using “repeat-backs” (68.2% vs 71.4% correct on the test). Low cost, non-technological solutions to improving consent form comprehension are spreading, such as including a comprehension quiz or having both the research coordinator and the potential subject initial each page before proceeding. Some research projects utilize an online video or slide deck describing the study, in some cases including the actual consent form.

In the present study, freedom to stop participating at any time was the only question where participants exceeded an 80% correct threshold. Part 1 and Part 2 subjects assigned to the paper consent scored below 50% on multiple questions (four questions with Part 1 and three questions with Part 2). Interestingly, research professionals did not do particularly well with the more technical questions regarding quantitative thermal sensory testing. Overall, the research professionals performed almost exactly the same as the participants drawn from clinic waiting rooms with both the iPad and paper versions.

How should the results of the current study be interpreted and applied? The central aim of this randomized study was to compare an interactive, tablet-based presentation of a clinical research consent form with a paper version of the same consent on comprehension of study procedures and risks. The interactive system was tested in a simulated situation where research professionals and patients were asked to pretend they had cancer and were considering entering a research study. They knew that comprehension was going to be tested after a time delay. This likely added to the length of time spent reviewing the consent form compared to the earlier studies of Baren, McNutt, and Lee [3]–[5]. Among both research professionals and patients, next-day comprehension was better in subjects randomized to the interactive iPad consent form. The iPad group received study information in multiple ways; via a brief video before viewing the consent form, and followed by a 7-item quiz where incorrect responses resulted in seeing relevant text from the consent form before trying to answer again. For those online survey questions that had been a part of the quiz presented at the end of the iPad version of the consent form, the probability of a correct answer was higher in both Part 1 and Part 2 participants. However, simple repetition accounts for only part of the improvement as participants randomized to the iPad also performed better on questions that hadn’t been part of the quiz. The iPad participants spent more time with the device, but the amount of time spent reviewing the actual consent document was actually shorter (13 minutes for paper, 11.4 minutes on the iPad). The introductory video about the study was relatively brief at just under six minutes in length, and a few subjects chose to view it twice. The quiz can be time consuming if a participant makes multiple incorrect choices on the same question. If the interactive consent were to be used in an actual study, participants making too many errors would not be allowed to proceed further before reviewing the areas of misunderstanding with the investigator or appropriate staff member. The errors could guide the discussion toward specific areas of the study.

Although the interactive consent system used adds cost to conducting a study, features such as automatic archiving of consent documentation are useful independent of the enhanced consenting process. An interactive consent process on a tablet – such as an iPad – would allow the patient to go through the consent process in the office and even take the tablet home. For long term studies or therapies requiring multiple visits, the iPad is applicable to research and clinical practice as the portal for completing symptom ratings, reporting outcomes and adverse events, and completing questionnaires. In summary, this study demonstrates that combining an introductory video, standard consent language, and an interactive quiz on a tablet-based system improves comprehension of procedures and risks.
